# Balloon enteroscope insertion over an endoscopic nasobiliary drainage tube in patients with surgically altered anatomy

**DOI:** 10.1055/a-2658-0271

**Published:** 2025-08-08

**Authors:** Yoshihiro Goda, Kuniyasu Irie, Yuto Matsuoka, Tomomi Hamaguchi, Hideyuki Anan, Yoshimasa Suzuki, Shin Maeda

**Affiliations:** 1 218758 Gastroenterology, Yokohama City University Hospital, Yokohama, Japan; 2 Gastroenterology Division, Yokohama City University, School of Medicine, Yokohama, Japan

Scope insertion over an endoscopic nasobiliary drainage (ENBD) tube can facilitate the exchange to internal drainage without the need for selective guidewire reinsertion 1 2 3 . During the procedure, an assistant pushes or pulls the ENBD tube to prevent distal migration of the tube tip ( Fig. 1 ). However, insertion of a balloon enteroscope over an ENBD tube in patients with surgically altered anatomy is challenging because of the insufficient length of the tube relative to the enteroscope, which precludes positional adjustment by the assistant. Here, we report a case of successful balloon enteroscope insertion over an ENBD tube followed by the exchange of the tube with a plastic stent.

**Fig. 1:**
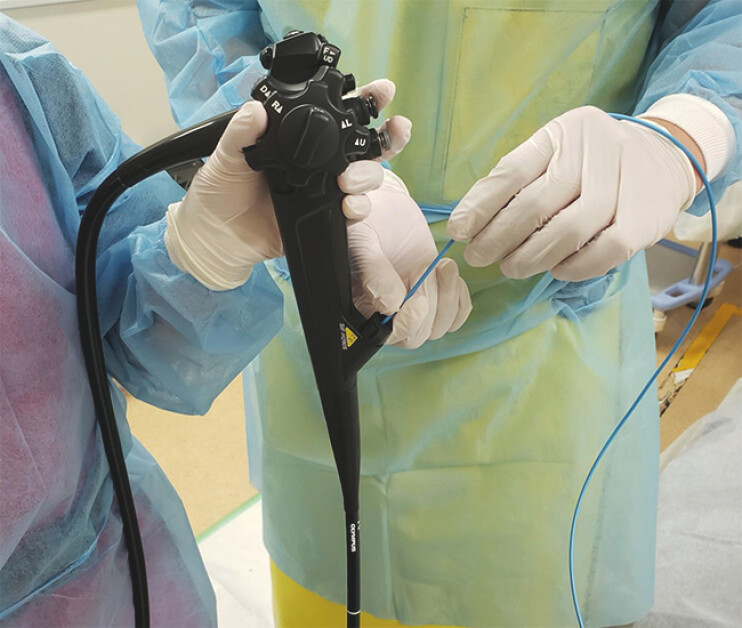
During scope insertion, the assistant adjusts the endoscopic nasobiliary drainage tube to prevent distal migration of the tube tip.

A 55-year-old man who had undergone pancreaticoduodenectomy with Billroth II reconstruction for pancreatic neuroendocrine neoplasm was diagnosed with afferent loop obstruction on computed tomography ( Fig. 2 ). Balloon enteroscopy-assisted endoscopic retrograde cholangiopancreatography (ERCP) with short-type single-balloon enteroscope (SIF-H290S; Olympus Medical Systems, Tokyo, Japan) was performed and a 7-Fr ENBD tube (Liguory Nasal Biliary Drainage Set; Cook Medical, Tokyo, Japan) was placed ( Fig. 3 ).

**Fig. 2:**
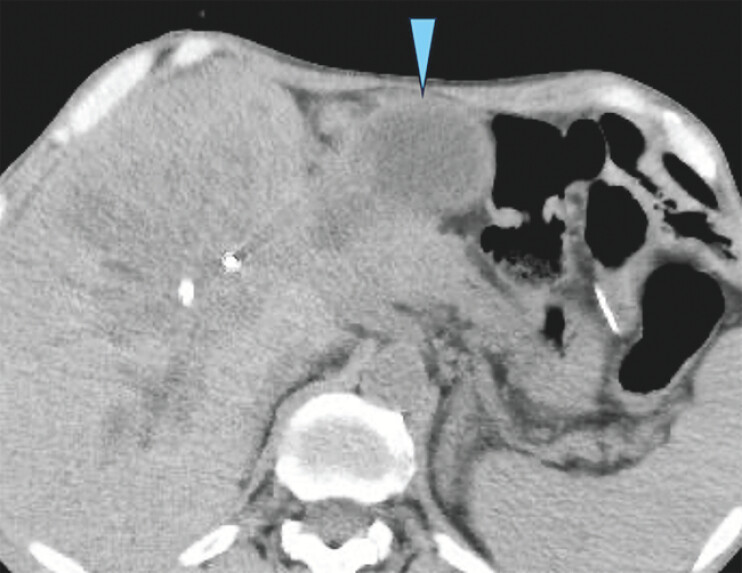
Computed tomography showing afferent loop obstruction (arrowhead).

**Fig. 3:**
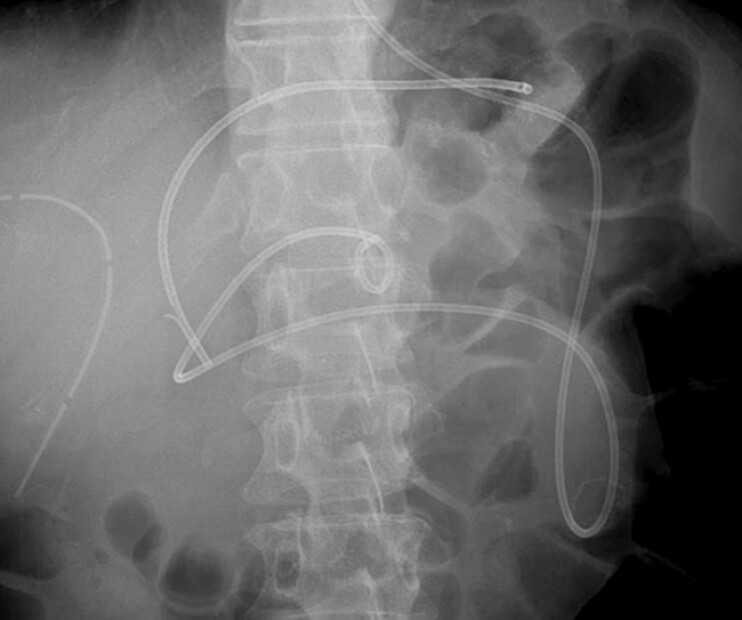
The endoscopic nasobiliary drainage tube (Liguory Nasal Biliary Drainage Set; Cook Medical, Tokyo, Japan) was placed for afferent loop obstruction.

After resolution of the inflammation, balloon enteroscope-assisted ERCP was repeated to exchange the ENBD tube for internal drainage. The ENBD tube was repositioned from the nostril to the mouth 4 , and the tip was grasped with a snare and pulled into the accessory channel of the enteroscope ( Fig. 4 ). The balloon enteroscope was then advanced over the ENBD tube while the assistant adjusted tube positioning during insertion ( Fig. 1 , Video 1 ). The enteroscope successfully reached the duodenal stricture. A guidewire was inserted through the ENBD tube, which was then removed. Contrast-enhanced imaging confirmed duodenal stenosis ( Fig. 5 **a** ). A 7-Fr plastic stent (Through & Pass double pigtail; Gadelius, Tokyo, Japan) was successfully placed ( Fig. 5 **b** ).

**Fig. 4:**
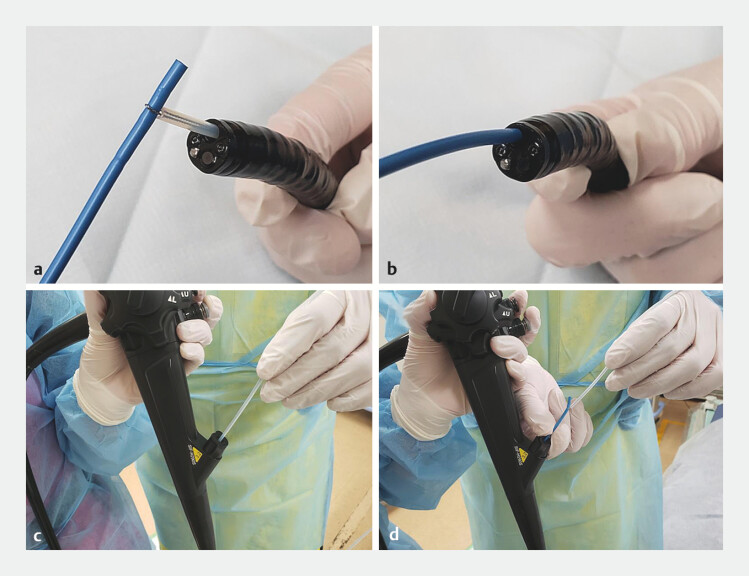
Tube application. **a** The endoscopic nasobiliary drainage tube tip was grasped with a snare. **b–d** The tube was pulled into the accessory channel of the enteroscope, enabling the assistant to adjust tube position during scope insertion.

**Fig. 5:**
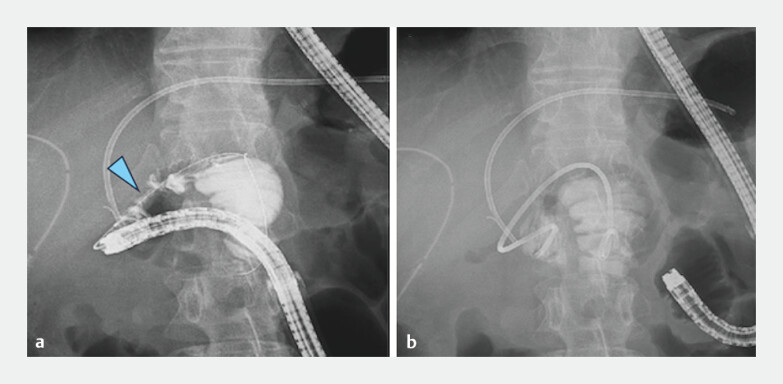
Contrast-enhanced imaging. **a** Duodenal stenosis (arrowhead) was confirmed. **b** A 7-Fr plastic stent (Through & Pass double pigtail; Gadelius, Tokyo, Japan) was successfully placed.

Scope insertion over an endoscopic nasobiliary drainage tube was feasible and useful in a patient with surgically altered anatomy.Video 1

To the best of our knowledge, this is the first report of balloon enteroscope insertion over an ENBD tube in a patient with surgically altered anatomy.

Endoscopy_UCTN_Code_TTT_1AP_2AD

## Notes

Endoscopy E-Videos https://eref.thieme.de/e-videos
*E-Videos* is an open access online section of the journal *Endoscopy* , reporting on interesting cases and new techniques in gastroenterological endoscopy. All papers include a high-quality video and are published with a Creative Commons CC-BY license. Endoscopy E-Videos qualify for HINARI discounts and waivers and eligibility is automatically checked during the submission process. We grant 100% waivers to articles whose corresponding authors are based in Group A countries and 50% waivers to those who are based in Group B countries as classified by Research4Life (see: https://www.research4life.org/access/eligibility/ ). This section has its own submission website at https://mc.manuscriptcentral.com/e-videos .
